# Physical active lifestyle promotes static and dynamic balance performance in young and older adults

**DOI:** 10.3389/fphys.2022.986881

**Published:** 2022-08-17

**Authors:** Fabio Sarto, Martina Pizzichemi, Francesco Chiossi, Patrizia S. Bisiacchi, Martino V Franchi, Marco V Narici, Elena Monti, Antonio Paoli, Giuseppe Marcolin

**Affiliations:** ^1^ Department of Biomedical Sciences, University of Padova, Padua, Italy; ^2^ School of Human Movement Science, University of Padova, Padua, Italy; ^3^ Department of General Psychology, University of Padova, Padua, Italy; ^4^ LMU Munich, Munich, Germany

**Keywords:** sedentarism, exercise, aging, balance control, physical exercise

## Abstract

Although regular physical activity exposure leads to positive postural balance control (PBC) adaptations, few studies investigated its effects, or the one of inactivity, on PBC in populations of different age groups. Thus, this study investigated the impact of a physically active lifestyle on static and dynamic PBC in young and older adults. Thirty-five young physically active subjects (YA), 20 young sedentary subjects (YS), 16 physically active older adults (OA), and 15 sedentary older adults (OS) underwent a static and a dynamic PBC assessment. A force platform and an instrumented proprioceptive board were employed to measure the center of pressure (COP) trajectory and the anteroposterior oscillations, respectively. In static conditions, no significant differences were detected among groups considering the overall postural balance performance represented by the area of confidence ellipse values. Conversely, the YA highlighted a higher efficiency (i.e., lower sway path mean velocity) in PBC maintenance compared to the other groups (YA vs OA: *p* = 0.0057, Cohen’s d = 0.94; YA vs OS *p* = 0.043, d = 1.07; YA vs YS *p* = 0.08, d = 0.67). OS exhibited an overall worse performance in dynamic conditions than YA and YS. Surprisingly, no differences were found between YS and OA for all the static and dynamic parameters considered. In conclusion, our results suggest that a physically active lifestyle may promote static and dynamic balance performance in young and older adults, thus with potentially positive effects on the age-related decline of postural balance performance. Dynamic PBC assessment seems more sensitive in detecting differences between groups than the static evaluation.

## Introduction

Postural balance control (PBC) is a fundamental ability to accomplish every motor task in daily life and sports (Zemková, 2014; Paillard, 2017a). This ability relies on the efficiency of the integrated activity of the visual, vestibular, and proprioceptive systems and can be influenced by regular sports practice and motor experience (Paillard, 2017a).

Although acute physical activity (PA) has been shown to impair PBC by altering the effectiveness of sensory inputs and motor output (Marcolin et al., 2019), regular PA exposure can lead to positive PBC adaptations (Lelard and Ahmaidi, 2015; Paillard, 2017a). For instance, it is well established that specific balance training effectively improves static and dynamic PBC, under stable and unstable conditions, with eyes open and closed, both in young and older adults (DiStefano et al., 2009; Lesinski et al., 2015b). Moreover, a dose-response relationship has been previously observed in a meta-analysis evaluating the effects of balance training on PBC (Lesinski et al., 2015a). “Well-being” physical activities (e.g., Tai Chi, Yoga, and Qigong), strength training, and sports activities also improve PBC (Lelard and Ahmaidi, 2015; Paillard, 2017a). Interestingly, in older populations, positive PBC adaptations can also occur by performing simple domestic or daily PA tasks, such as the regular practice of stair climbing and brief walking (Brooke-Wavell et al., 1998; Paillard et al., 2005). The positive associations between PA levels (assessed using self-reported questionnaires or accelerometers) and PBC performance have been observed in different populations (Persson et al., 2016; Morimoto et al., 2019) and appear to be driven by a complex series of adaptations involving the sensory, central and motor components of the postural function (Paillard, 2017a).

PBC is a multifactorial motor skill (Pollock et al., 2000) in which various systems (i.e., visual, somatosensory, vestibular, and musculoskeletal) are involved (Horak et al., 1989; Takakusaki et al., 2017). Since normal aging is accompanied by a physiological deterioration of the integrity of these systems, it is not surprising that reductions in PBC performance have been observed in older populations (Hytönen et al., 1993; Baloh et al., 1994; Onambele et al., 2006). However, the PBC impairments detected in advanced age may be related to the effects of aging per se and the increased tendency among older people to become sedentary (McPhee et al., 2016). Surprisingly, only few studies investigated the effects of regular PA exposure (i.e., physically active lifestyle through structured recreational physical activity practice) and sedentarism on PBC in young and older adults. These showed that highly aerobic-trained older adults (i.e., masters runners) were not spared from the age-associated decline in postural stability, despite a superior performance compared to non-athletic peers (Leightley et al., 2017).

PBC has generally been assessed through static posturography measuring, with force platforms, the center of pressure (CoP) displacements. However, there is mounting evidence that static PBC assessment alone is not sufficiently challenging to assess the overall postural function (Ross and Guskiewicz, 2004; Petró et al., 2017). On this point, some authors (Ringhof and Stein, 2018; Rizzato et al., 2021) extended this traditional perspective considering balance as a general ability, highlighting the concept that dynamic balance tests are also necessary and not interchangeable. For instance, previous studies suggested employing dynamic rather than static tests to detect PBC impairments resulting from previous injury (Ross and Guskiewicz, 2004; Sarto et al., 2019) or to study the impact of acute physical exercise on PBC (Marcolin et al., 2019). Nonetheless, although dynamic PBC may provide more accurate insights into the postural function, it is still poorly studied, and little is known about how PA exposure could influence dynamic PBC. Moreover, it is currently poorly investigated in clinical practice whether adding a dynamic PBC assessment for older adults could provide additional information on physical function.

In light of these previous works, we aimed to deepen, with a cross-sectional study, the effects a physically active lifestyle (i.e., structured recreational physical activity practice) could have on static (SPBC) and dynamic (DPBC) PBC performance comparing young and older adults. We hypothesized that older adults would show an overall impairment of PBC compared to young adults; however, we expected that a physically active lifestyle would at least partially slow down the detrimental effects of aging on PBC.

## Methods

### Participants

86 participants (19 females and 67 males) volunteered for this study. We recruited 35 young physically active subjects (YA; age: 26.08 ± 4.47 years, height: 1.83 ± 0.08 m, body mass: 75.9 ± 9.9 kg), 20 young sedentary subjects (YS; age: 24.6 ± 1.46 years, height: 1.76 ± 0.07 m, body mass: 70.95 ± 10 kg), 16 physically active older adults (OA; age: 70.12 ± 3.44 years, height: 1.65 ± 0.08 m, body mass: 71.5 ± 7.8 kg) and 15 sedentary older adults (OS; age: 70.93 ± 6.25 years, height: 1.63 ± 7.2 m, body mass: 72.5 ± 8.8 kg). The YA trained 3 to 5 times per week and were competitive team players or endurance athletes. The OA practiced structured physical activity (dance, yoga, fitness, and other light aerobic activities) at least 2 times/week (on average ∼5/h week) for at least 5 years. At the time of the study, the YS and OS had not been involved in any structured form of PA for at least 5 and 15 years, respectively. Inclusion criteria for all participants included the absence of musculoskeletal injuries in the last 12 months and the active presence of neurological pathologies, sight, hearing, and vestibular disorders.

### Protocol

The study was conducted from March to July 2019. Before data collection, all subjects were instructed about the experimental procedures and signed informed consent. This study was carried out following the Declaration of Helsinki. The study design was approved by the Ethics Committee of the Department of Biomedical Sciences, University of Padova, Italy.

A sub-set of the present SPBC and DPBC datasets presented in this work has been presented elsewhere (Sarto et al., 2020; Marcolin et al., 2021). Participants have been asked to visit the laboratory twice. In the first visit, each participant underwent a 5 min familiarization session on a proprioceptive board (see below). After 1 week, the participants attended the testing session.

### Static postural balance assessment

A bipodalic static balance test was carried out on a force platform at a sampling rate of 100 Hz (AMTI BP 400600, AMTI, Watertown, United States). Subjects were instructed to stand on the platform with arms relaxed along their sides, heels aligned, and feet forming an angle of 30° (Kapteyn et al., 1983). The participants were barefoot and had to gaze at a target placed on a wall at a 1 m distance. They performed two trials of 40 s. The static balance performance was evaluated by two parameters averaged over the two trials: the area of the confidence ellipse (cm^2^), where the CoP has a 95% chance to fall within, and the CoP sway path mean velocity (cm/sec).

### Dynamic postural balance assessment

The DPBC was assessed throughout an instrumented proprioceptive board enabling oscillations along one single axis (i.e., allowing only anterior-posterior oscillations), as described previously (Sarto et al., 2020). Two reflective markers were placed on the right side of the platform. Their trajectory was recorded with a six-camera motion capture system at 120 Hz (OptiTrack, NaturalPoint^®^, Corvallis, OR, United States). Subjects were asked to stand on the platform aligning the mid-point of each foot (i.e., the half of the distance between the medial malleolus and the basis of the first metatarsus) with the mid-line of the platform. Participants were instructed to keep the board parallel to the floor as much as possible. Each subject performed two trials of 40 s. The post-processing analysis was performed with the software Smart Tracker (BTS, Milan, Italy) and Smart Analyzer (BTS, Milan, Italy) to reconstruct the angular oscillations of the platform over time, obtained from the trajectory of the two markers applied on its edge. The dynamic balance performance was assessed by the following parameters (Sarto et al., 2020): 1) the integral of the angle-time (deg·s) curve (Full Balance, FB), 2) the time (s) each subject was able to maintain the platform between +4° and −4° (Fine Balance, FiB) and 3) between +8° and −8° (Gross Balance, GB). Small values in the FB reflect a superior postural performance, while for the FiB and GB, the higher the value, the better the postural performance. As for the static condition, the value of each parameter was averaged over the two trials.

### Statistical analysis

An *a priori* power analysis was performed with G*Power3.1.9.2 software. Using the one-way ANOVA test, setting the alpha error at 0.05, the Power at 0.80, and comparing four groups with a large effect size (f = 0.40), we obtained a total sample of 76 participants. The normality of each dataset was evaluated by the Shapiro-Wilk normality test. All the considered parameters did not pass the normality tests. Thus, a natural logarithm (Ln) transformation was employed, and the normality distribution was tested again. The static parameters and the FB datasets passed the normality test after transformation, while GB and FiB datasets did not. Thus, for these two latter parameters, a non-parametric statistic was applied. One-way analysis of variance was carried out to compare the performance among groups for the area of the confidence ellipse, sway path mean velocity and FB parameters. Post-hoc comparisons were evaluated using Tukey’s test. Kruskal–Wallis non-parametric test with Dunn’s multiple comparison test was employed for FiB and GB. The level of significance was set at *p* < 0.05. Data analysis was performed with the software package JASP (Version 0.15. University of Amsterdam, Netherlands). Cohen’s d was calculated with G*Power 3.1.9.2 software and interpreted as trivial (0.00–0.19), small (0.20–0.59), moderate (0.60–1.19), large (1.20–1.99), and very large (>2.00) (Hopkins et al., 2009). The effect size for the Kruskal–Wallis test as the eta squared based on the H-statistic was computed with R Studio 1.4 (R Core Team, 2018; rstatix (1.5.1) package).

## Results

**FIGURE 1:**
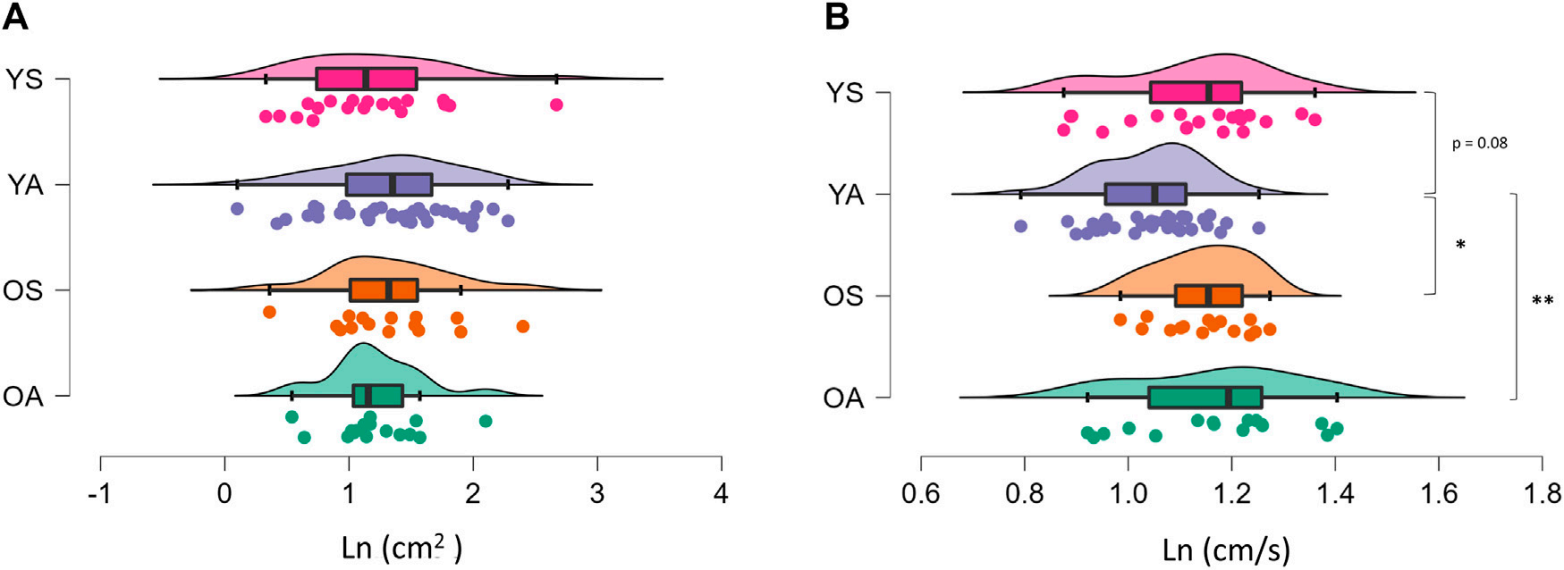
Raincloud plot of the differences among groups concerning the static postural balance control. The ‘cloud’ illustrates data distribution, while the ‘rain’ the jittered raw data. **(A)** area of the confidence ellipse; **(B)** sway path mean velocity. *p < 0.05; **p < 0.01. YA: physically active young adults; YS: sedentary young adults; OA: older active adults; OS: older sedentary adults.

**FIGURE 2:**
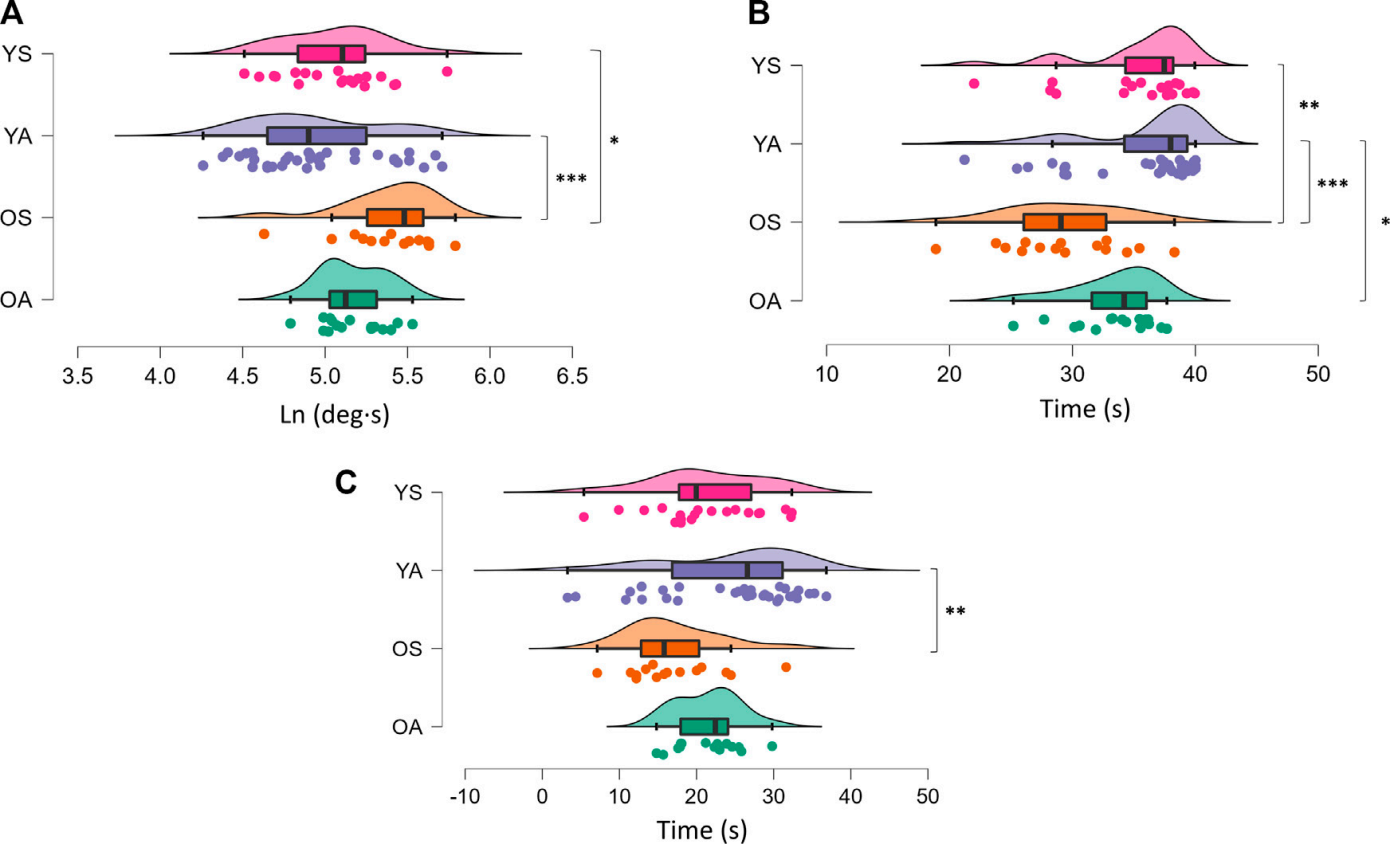
Raincloud plot of the differences among groups concerning the dynamic postural balance control. The ‘cloud’ illustrates data distribution, while the ‘rain’ the jittered raw data. **(A)** Full balance; **(B)** Gross balance; **(C)** Fine balance. *p < 0.05; **p < 0.01; ***p < 0.001. YA: physically active young adults; YS: sedentary young adults; OA: older active adults; OS: older sedentary adults.

Values of all the static and dynamic parameters are presented in Table 1. In the SPBC assessment, no significant differences among groups were detected in the area of confidence ellipse (Figure 1A). Differently, sway path mean velocity was significantly altered (F3,82 = 0.4575; *p* = 0.0119; η_p_^2^ = 0.158) among groups (Figure 1B). Tukey’s post-hoc analysis showed an increased sway path mean velocity for OA (*p* = 0.0057; d = 0.94) and OS (*p* = 0.043; d = 1.07) compared to YA, with a trend towards statistical significance also observed between YS and YA (*p* = 0.08; d = 0.67). In dynamic conditions, all parameters were affected by the group, with changes in FB (F3,82 = 6.669; *p* = 0.0004; η_p_^2^ = 0.196) (Figure 2A), GB (*p* < 0.0001; η_p_^2^ = 0.253) (Figure 2B) and FiB (*p* = 0.0142; η_p_^2^ = 0.125) (Figure 2C). Post-hoc analysis revealed an impaired FB in OS compared to YA (*p* = 0.0002; d = 1.28) and YS (*p* = 0.026; d = 1.08). Moreover, the YA exhibited higher FiB values than OS (*p* = 0.004; d = 0.92). Finally, the GB showed a worse postural performance of the OS with respect to YA (*p* = 0.0001; d = 1.29) and YS (*p* = 0.005; d = 1.2), with a significant difference also observed between YA and OA (*p* = 0.022; d = 0.56).

**TABLE 1:** Results of the static and dynamic tests. Data are presented as Means and Standard deviations.

	YA	YS	OA	OS
Area of the confidence ellipse (cm^2^)	1.58 (0.78)	1.46 (1.07)	1.32 (0.56)	1.57 (0.87)
Sway path mean velocity (cm/s)	2.86 (0.28)	3.12 (0.43)	3.26 (0.51)	3.15 (0.27)
Full balance (deg∙s)	151.8 (64.96)	165.4 (53.16)	179.6 (36.5)	226.7 (57.1)
Gross balance (s)	35.87 (5.09)	35.26 (4.8)	33.41 (3.52)	29.29 (5.09)
Fine balance (s)	24.27 (9.11)	21.23 (7.37)	21.46 (4.13)	17.06 (6.21)

## Discussion

With the present cross-sectional study, we aimed to compare the effects of a physically active lifestyle on static and dynamic PBC performance in young and older adults. The main findings were the following: (i) similar PBC performance was observed between YS and OA for all the static and dynamic parameters considered; (ii) YA exhibited a better PBC efficiency in static conditions compared to the other groups; (iii) OS were the group that performed worse in the dynamic task.

In static conditions, no differences were noted among groups considering the area of the confidence ellipse, while YA showed a lower sway path mean velocity (i.e., a lower mean velocity of the CoP) than all the other groups. The area of the confidence ellipse is the most commonly employed measure of SPBC, and it is considered a proxy of the overall static postural performance (Paillard and Noé, 2015), while the sway path mean velocity represents the neuromuscular activity needed to preserve balance, and thus the efficiency in the maintenance of PBC (Paillard and Noé, 2015). Therefore, our results showed that YA had greater postural balance efficiency with respect to YS, OA, and OS in static conditions but with no differences in the overall static postural performance (i.e., area of the confidence ellipse values). The reason for the unchanged area of the confidence ellipse among groups may be related to the static nature of the task, which has already been considered not sufficiently challenging for the postural system in healthy subjects (Petró et al., 2017), and thus not sensitive enough to detect differences among groups (Ross and Guskiewicz, 2004; Marcolin et al., 2019; Sarto et al., 2019). Instead, the increased SPBC efficiency in YA may be explained through different mechanisms. Despite the test employed, a superior SPBC has been generally observed in young versus old populations (Baloh et al., 1994; Onambele et al., 2006). Changes in neural control (Baudry, 2016), proprioception (Horak et al., 1989; Henry and Baudry, 2019), and muscle-tendon characteristics (Onambele et al., 2006) have been considered the principal physiological mechanisms underpinning the postural balance alterations occurring in older adults. Nonetheless, our findings on SPBC showed no differences between YS and older groups, suggesting that aging per se cannot explain the superior efficiency in SPBC of YA. Thus, we can hypothesize that YA performed better in SPBC than older adults, likely due to their prolonged PA practice that may have induced positive changes in the sensory, central, and motor components of the postural function (Paillard, 2017a). The positive SPBC adaptations that occurred in YA could also explain their increased efficiency in SPBC compared to YS, in agreement with previous works that found superior SPBC in young subjects practicing sports activity than aged-matched sedentary controls (Matsuda et al., 2008; Herpin et al., 2010).

In dynamic conditions, our findings revealed that OS exhibited an overall impairment of PBC. Indeed, OS displayed a worse FB and GB than the young cohorts and a worse FiB than YA. Although no differences were detected between OA and all the other groups, in a previous study (Marcolin et al., 2021), including a sub-set of the present sample, significant differences comparing only OA and OS emerged for all the DPBC parameters with large effects sizes.

As previously discussed, the aging process leads to a physiological/pathophysiological deterioration of the different systems involved in PBC (Horak et al., 1989; Baudry, 2016; Henry and Baudry, 2019), which partially explains the reduced DPBC performance of the OS compared to YA and YS. However, prolonged PA exposure in OA seems to guarantee more robust safeguarding from these age-related alterations. This finding is in line with previous studies showing a superior DPBC during tests based on underfoot perturbations in physically active older adults (Perrin et al., 1999) and masters athletes (Brauer et al., 2008) compared to sedentary peers. Besides the mechanisms mentioned above by which PA positively influences PBC (Paillard, 2017a), in dynamic conditions, compensatory postural actions are also facilitated by increased lower-extremity muscle power (Paillard, 2017b). Moreover, in a recent study from our laboratory, we found an association between neuromuscular junction damage and dynamic balance impairment (Marcolin et al., 2021). Since physical exercise is well known to promote both muscle power (Ramsey et al., 2021) and neuromuscular junction stability (Pratt et al., 2020) in older adults, PA-induced preservation of muscle power and neuromuscular junction health status in OA may contribute to explaining our findings in DPBC.

Some limitations must be acknowledged. First, due to the cross-sectional nature of the study, we cannot imply that the observed differences in PBC have been induced exclusively by a physically active lifestyle, but other confounding factors might exist. A second limitation is that we could not investigate the intensity of the participant PA and the history of PA during the youth and adulthood of our older adults, but only their relatively recent PA experience.

## Conclusion

In this study, we support the concept that a physically active lifestyle may positively influence PBC in young and older individuals. Surprisingly, no differences were observed in static and dynamic PBC performance comparing YS and OA. Since DPBC assessment seems more sensitive in detecting differences between groups than the static evaluation, we recommend that dynamic measures of PBC accompany the assessment of SPBC in older adults.

## Data Availability

The original contributions presented in the study are included in the article/Supplementary Material, further inquiries can be directed to the corresponding author.
